# A Robust Immuno-Prognostic Model of Non-Muscle-Invasive Bladder Cancer Indicates Dynamic Interaction in Tumor Immune Microenvironment Contributes to Cancer Progression

**DOI:** 10.3389/fgene.2022.833989

**Published:** 2022-06-03

**Authors:** Xiaomeng Sun, Huilin Xu, Gang Liu, Jiani Chen, Jinrong Xu, Mingming Li, Lei Liu

**Affiliations:** ^1^ Institutes of Biomedical Sciences and School of Basic Medical Sciences, Fudan University, Shanghai, China; ^2^ Research Institute, GloriousMed Clinical Laboratory Co., Ltd., Shanghai, China; ^3^ Department of Pharmacy, Second Affiliated Hospital of Naval Medical University, Shanghai, China; ^4^ Department of Electronic Engineering, Taiyuan Institute of Technology, Taiyuan, China

**Keywords:** non-muscle-invasive bladder cancer, tumor immune microenvironment, tumor progression, collagen family, cancer-associated fibroblasts

## Abstract

Non-muscle-invasive bladder cancer (NMIBC) accounts for more than 70% of urothelial cancer. More than half of NMIBC patients experience recurrence, progression, or metastasis, which essentially reduces life quality and survival time. Identifying the high-risk patients prone to progression remains the primary concern of risk management of NMIBC. In this study, we included 1370 NMIBC transcripts data from nine public datasets, identified nine tumor-infiltrating marker cells highly related to the survival of NMIBC, quantified the cells’ proportion by self-defined differentially expressed signature genes, and established a robust immuno-prognostic model dividing NMIBC patients into low-risk versus high-risk progression groups. Our model implies that the loss of crosstalk between tumor cells and adjacent normal epithelium, along with enriched cell proliferation signals, may facilitate tumor progression. Thus, evaluating tumor progression should consider various components in the tumor immune microenvironment instead of the single marker in a single dimension. Moreover, we also appeal to the necessity of using appropriate meta-analysis methods to integrate the evidence from multiple sources in the feature selection step from large-scale heterogeneous omics data such as our study.

## Introduction

Bladder cancer contributed to 573,278 new cases and 212,536 deaths worldwide (Sung et al., 2021) in 2020. It is one of the cancers with the most longitudinal costs and consumed resources. Approximately 70–75% of newly diagnosed primary bladder cancers are non-muscle-invasive bladder cancer (NMIBC) (Lenis et al., 2020; Ottley et al., 2020). Up to 21–53% of them eventually progress to life-threatening muscle-invasive bladder cancer (MIBC) (Cookson et al., 1997; van den Bosch and Alfred Witjes, 2011), depending on the stage and grade. Identifying the NMIBC patients with a high progression potential at the early treatment stage remains the primary object of bladder cancer clinical practice.

Several risk classification frameworks have been suggested and applied in NMIBC risk management. European Association of Urology (EAU) prognostic factor risk groups updated the EAU NMIBC Guidelines Panel in 2021 by dividing NMIBC patients into four risk groups: low-, intermediate-, high-, and a new, very high-risk group, with the probability of progression at 5-year of <1%, 3.6–4.9%, 9.6–11%, and >40% (Sylvester et al., 2021). Clinicopathological features employed in the panel included: tumor stage, the World Health Organization (WHO) 1973 or 2004/2016 grade, concomitant carcinoma *in situ* (CIS or Tis), number of tumors, tumor size, and age. American Urological Association (AUA) and Society of Urologic Oncology (SUO) also amended the AUA/SUO Joint Guideline in 2020 by classifying NMIBC patients into low-, intermediate-, and high-risk groups (Chang et al., 2016; Chang et al., 2020). Apart from the clinical features used in the EAU Panel, AUA risk stratification also took variant histology, preceding recurrent disease, Bacillus Calmette-Guerin (BCG) treatment failure, and involvement of prostatic urethral into consideration. Although such frameworks essentially help the risk management of NMIBC patients and are readily used in bedside patient care, a more precise solution is always in need.

To fulfill the need, molecular subtyping and gene expression modeling based on the omics analysis have become mainstream in clinical decision support scenarios like diagnosis, treatment response prediction, and prognostic stratification. The UROMOL project, a European multicenter prospective study of NMIBC spanning from 2008 to date, identified high-risk class 2a tumors at the transcriptomic level and high-risk class GC3 tumors at the genomic level (Lindskrog et al., 2021). They also revealed that higher immune cell infiltration strongly correlated with lower recurrence rates. However, the association between immune cell infiltration and cancer progression remained unknown. Since there were too few progression events for evaluating its effect on progression-free survival (PFS), Zheng and colleagues developed an immune prognostic signature (IPS) based on 14 overall survival (OS) associated immune genes. Then they proved that high-risk patients assessed by the IPS score had worse OS than those with low-risk scores in validation datasets (Zheng et al., 2020). Ottley et al. studied the correlations between 11 antibodies relating to molecular subtypes or epithelial-to-mesenchymal transition (EMT) and prognosis in high-risk non-muscle-invasive (HGT1) bladder cancer. They found that both stromal tumor-infiltrating lymphocyte (sTIL) levels in noninvasive papillary urothelial carcinoma areas and increased expression of the luminal markers FOXA1 and SCUBE2 are significantly associated with better disease-free survival (DFS), but no EMT markers showed any trend. They suggested that molecular subtype markers, rather than EMT markers, might be preferable to study biomarkers of HGT1 urothelial carcinoma (Ottley et al., 2020). Rouanne et al. focused on stromal lymphocyte infiltration by evaluating the percentage of stromal area infiltrated by mononuclear inflammatory cells over the total intratumoral stromal area (Rouanne et al., 2019). Similarly, a high density of stromal TILs was associated with the tumor invasion depth in pT1 NMIBC, implying tumor aggressiveness was associated with an increased adaptive immune response, but no association between the level of TILs and survival outcome was observed. A clear clue has shown that the activated tumor immune microenvironment (TIME) could prevent NMIBC tumors from progressing. However, additional integration and refinement of these findings are required to provide a robust immuno-prognostic model for predicting progression in NMIBC patients.

In this study, we reported an integrated analysis using a total of 1370 transcriptome data of NMIBC patients from nine public datasets. Candidate tumor-infiltrating immune cells relating to the well-established prognostic risk factors and survival were filtered by a non-weighted voting system of six deconvolution methods and the survival analysis. Differentially expressed genes (DEGs) representing the candidate immune cells were identified. We used the selected DEGs as predefined signature genes in the single-sample gene set enrichment analysis (ssGSEA) to achieve unbiased quantification of the tumor-infiltrating immune cells. Finally, we developed a robust immune-prognostic model based on the immune cell matrix for evaluating the progression of NMIBC patients.

## Materials and Methods

### Transcriptomic Profiles Analyzed

We searched for public datasets using combined keywords of “NMIBC”, “expression profile”, and “human” through GEO (Barrett et al., 2013), ArrayExpress (Athar et al., 2019), and PubMed^®^ databases. Exclusion criteria of ineligible datasets were as follows: 1) datasets lacking cancer grade or TNM stage metadata; 2) datasets with only expression profiles of muscle-invasive bladder cancer (MIBC) samples; 3) datasets providing only processed data with negative expression values. Then we de-duplicated the same samples collected from multiple sources. Notably, our study allowed for the inclusion of datasets sequenced by RNA-Seq and microarray platforms. We also allowed sampling of tumors from both primary and recurrent lesions.

### Deconvolution of Tumor-Infiltrating Immune Cells

We employed six in silico deconvolution methods to estimate cell composition in 1370 human transcriptome data. The xCell (Aran et al., 2017) performed an enrichment analysis of 64 immune and stromal cell types, illustrating whether a particular type of cell was present. The immunedeconv (Sturm et al., 2019), an integrated deconvolution tool, implemented the other four cell-type quantification algorithms, including quanTIseq (Finotello et al., 2019), TIMER (Li et al., 2016), MCPCounter (Becht et al., 2016), and EPIC (Racle et al., 2017). Moreover, ESTIMATE (Yoshihara et al., 2013) was used to estimate combined immune, stromal, and ESTIMATE scores without giving any single cell-type proportion. In summary, we assessed 64 tumor-infiltrating immune cell scores and six immune infiltration biomarker scores for each processed sample. Names of the cells and biomarkers with their corresponding alias in the six deconvolution methods are provided in Supplementary Table S3.

### Correlations Between Clinicopathological Features and Immune Cells

To avoid methodological bias, we adopted an unweighted voting system to discover tumor-infiltrating immune cells significantly related to the well-established prognostic risk factors of NMIBC patients. In datasets providing age, sex, stage, grade, tumor size, European Organisation for Research and Treatment of Cancer (EORTC) risk score, and CIS in disease course status data, we compared the distribution of 64 tumor-infiltrating cell deconvolution scores across different levels of the risk factors. Student’s t-test and box plots were performed by the “ggplot2” (Wickham, 2016) package of R language (R Core Team, 2021). A cell type in a specific dataset deconvoluted by a particular algorithm with a false discovery rate (FDR) adjusted *p*-value of student’s t-test in more than two levels less than 0.05 was counted as one vote for the cell. All votes were categorized into 64 cell types to reveal the tumor immune microenvironment that would predict survival (Supplementary Table S4).

### Identification of Differentially Expressed Genes of Candidate Immune Cells

The “limma” (Ritchie et al., 2015) package of R language (R Core Team, 2021) was used to identify differentially expressed genes (DEGs) of each candidate immune cell type. Log2-transformed fold changes (log2FC), *p*-values, and FDR adjusted *p*-values of every “source dataset—deconvolution method—immune cell—gene name” sets are provided in Supplementary Table S5. Only genes with absolute log2FCs larger than one and FDR *p*-values less than 0.05 were defined as DEGs for corresponding cell types. Furthermore, we defined candidate “ cell-gene” combinations by the wFisher (Yoon et al., 2021) *p*-value in all evaluable sets, along with the number of datasets in which the combination was evaluable (Supplementary Table S6). The gene with a mean absolute log2FC larger than 0.2 for NK cells and 0.3 for other cells, a wFisher combined *p*-value less than 1.151e-6 (0.05/number of genes 43,440), and identified as significant DEGs in more than three databases were defined as representative gene of the immune cell. The “metapro” (Yoon et al., 2021) package in R (R Core Team, 2021) was used to calculate the combined wFisher *p* values.

### Identification of Immune-Cell-Specific DEGs Related to Survival

Faced with dozens to hundreds of DEGs representing one immune cell type, we further narrowed the list by conducting survival analyses in the Kaplan-Meier curve and the forest plot to remove genes that contribute less to survival risk. Divided by the median of candidate genes’ expression, we compared the PFS of E-MTAB-4321, DFS of GSE32894, and OS of GSE13507 in low expressed versus high expressed groups (results provided in Supplementary Table S7). The Kaplan-Meier curve was fitted by the “survfit” function and visualized by the “ggsurvplot” function. The forest plot was fitted by the “coxph” function and visualized by the “ggforest” function. DEGs with log-rank *p*-values of both analyses less than 0.05 and hazard ratios (HRs) of Cox’s proportional hazards models larger than 2.5 or less than 0.5 were defined as the final biomarker genes of the candidate immune cells. All survival analyses were implemented by the “survival” package (Therneau and Grambsch, 2000; Therneau, 2021) and visualized by the “ggplot2” (Wickham, 2016, 2) package in R (R Core Team, 2021). The “ComplexHeatmap” (Gu et al., 2016) package in R (R Core Team, 2021) was used to generate expression heatmaps of the final gene list.

### Gene Ontology and Pathway Enrichment of Candidate DEGs

We conducted Gene Ontology (GO) (Ashburner et al., 2000; Gene Ontology Consortium, 2021) and Kyoto Encyclopedia of Genes and Genomes (KEGG) (Kanehisa et al., 2021) pathway enrichment analyses of the selected immune-cell-specific DEGs by the “clusterProfiler” (Yu et al., 2012; Wu et al., 2021) package in R (R Core Team, 2021).

### Calculation of ssGSEA and Z-Score Based Cell Enrichment Scores

Inspired by previous studies (Barbie et al., 2009; Motzer et al., 2020), we employed two methods to evaluate the nine candidate immune cells using gene lists generated by previous steps. The ssGSEA analysis (Subramanian et al., 2005) was performed on the logged expression matrix by the “GSVA” (Hänzelmann et al., 2013) package in R (R Core Team, 2021), and z-score statistics were performed on the non-logged expression matrix by in-house scripts.

### Correlations Between Tumor-Infiltrating Immune Cell Score and Survival

Patients in each dataset were divided by the median of enriched immune cell scores into high and low immune infiltrated groups. Survival analyses and log-rank tests of PFS, DFS, and OS in high versus low immune cell infiltrated groups were conducted by the “survfit” function of the “survival” (Therneau and Grambsch, 2000; Therneau, 2021) package. Kaplan-Meier curves were visualized by the “ggsurvplot” function of the “ggplot2” (Wickham, 2016, 2) package in R (R Core Team, 2021). *p* values of both analyses and hazard ratios of high infiltrated groups are provided in Supplementary Table S8.

### Establishment of the Immuno-Prognostic Model

Using 454 samples from E-MTAB-4321 with evaluable PFS records, we randomly re-sampled 5000 times to build training and test sets in a 1:1 ratio. In each sampling scenario, we established a ridge regression model with an estimated enrichment score matrix of the nine tumor-infiltrating immune cells to predict the risk of progression. In each modeling process, tenfold cross-validation was used to select the optimal fitted model. The prediction performance of the models was evaluated by areas under curves (AUCs) of receiver operating characteristic curves (ROCs) in training and test sets. In R language (R Core Team, 2021), the “glmnet” (Friedman et al., 2010) package was used to build the models, and the “pROC” (Robin et al., 2011) package was used to visualize the results.

### Statistical Analysis

*p*-Values less than 0.05 were considered significant in this study unless otherwise specified.

## Results

### Summary of Datasets and Basic Workflow

**FIGURE 1:**
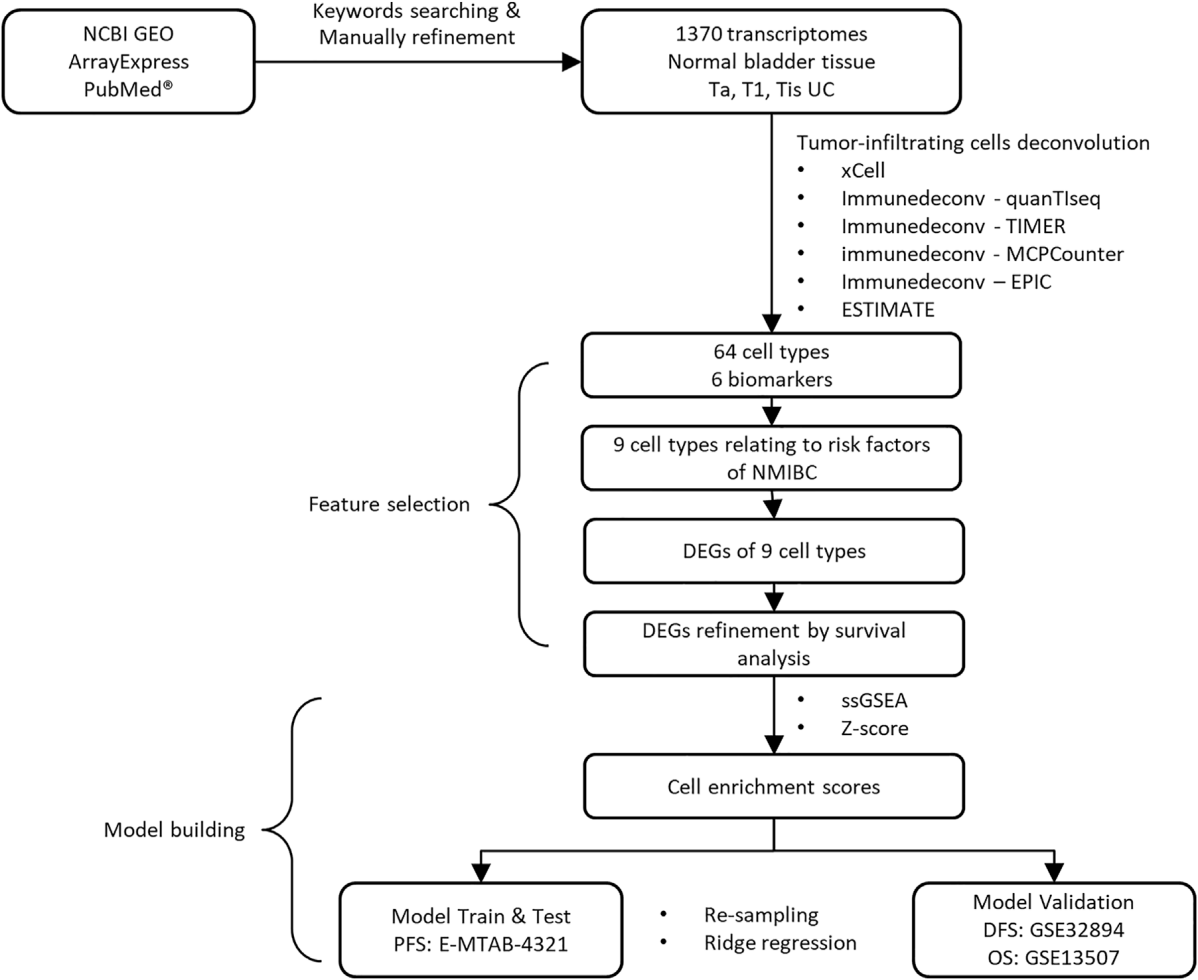
Overview of study design.

The study design and workflow to develop our model are illustrated in Figure 1. After keyword searching and manual refinement, we brought nine datasets into this study, including 1370 human transcriptome profiles spanning normal bladder tissues, Ta, T1, and CIS urothelial cancers. Metadata of all the datasets and clinicopathological information of all the samples are provided in Table 1; Supplementary Tables S1,S2.

**TABLE 1:** Demographic and disease characteristics of the 1,370 samples included in this study. Data are median (total number of assessable samples; range; IQR) or n (%). IQR: interquartile range. PFS: progression-free survival. DFS: disease-free survival. OS: overall survival.

Characteristics	Value
Age (years)	69 (862; 20–96; 61–76.5)
Age category (years)	—
20–60	198 (14%)
61–80	542 (40%)
> =80	122 (9%)
Not available	508 (37%)
Sex	—
Male	797 (58%)
Female	219 (16%)
Not available	354 (26%)
Tumor Stage	—
T0	91 (6%)
Ta	696 (51%)
Ta-T1	24 (2%)
T1	547 (40%)
CIS/Tis	12 (1%)
WHO 1973 Grade	—
G1	58 (4%)
G2	199 (15%)
G3	285 (21%)
G0/Gx/Not available	828 (60%)
WHO 2004–2016 Grade	—
Low	427 (31%)
High	289 (21%)
Not available	654 (48%)
CIS in the disease course	—
CIS-	472 (34%)
CIS+	103 (8%)
Not available	795 (58%)
Tumor size	—
<=3 cm	311 (23%)
>3 cm	83 (6%)
Not available	976 (71%)
EORTC risk score	—
0	286 (21%)
1	174 (13%)
Not available	910 (66%)
Recurrence	—
FALSE	127 (9%)
TRUE	57 (4%)
Not available	1186 (87%)
Progression beyond the T2 stage	—
FALSE	711 (52%)
TRUE	66 (5%)
Not available	593 (43%)
PFS (months)	33 (460; 0–74.9; 24–42.8)
Cancer-specific survival	—
FALSE	271 (20%)
TRUE	6 (∼0%)
Not available	1093 (80%)
DFS (months)	37.9 (173; 0.2–104.4; 21.2–60.2)
Vital status	—
FALSE	144 (11%)
TRUE	42 (3%)
Not available	1184 (86%)
OS (months)	55.3 (104; 2.1–137; 26.4–80.3)

With the 1370 transcriptomic profiles, we initially screened nine candidate immune cells associated with the well-established NMIBC prognostic risk factors and then identified the differentially expressed genes (DEGs) representing these cells by significance and differentiation. Using the DEGs’ expression matrix, we estimated the proportions of tumor infiltrated immune cells by the gene set enrichment analysis. Using the estimated immune cell score matrix, we established the immune-prognostic model by repeated random sampling, ridge regression modeling, and optimal cutoff confirming.

### Tumor-Infiltrating Immune Cells Related to Key NMIBC Prognostic Factors

**FIGURE 2:**
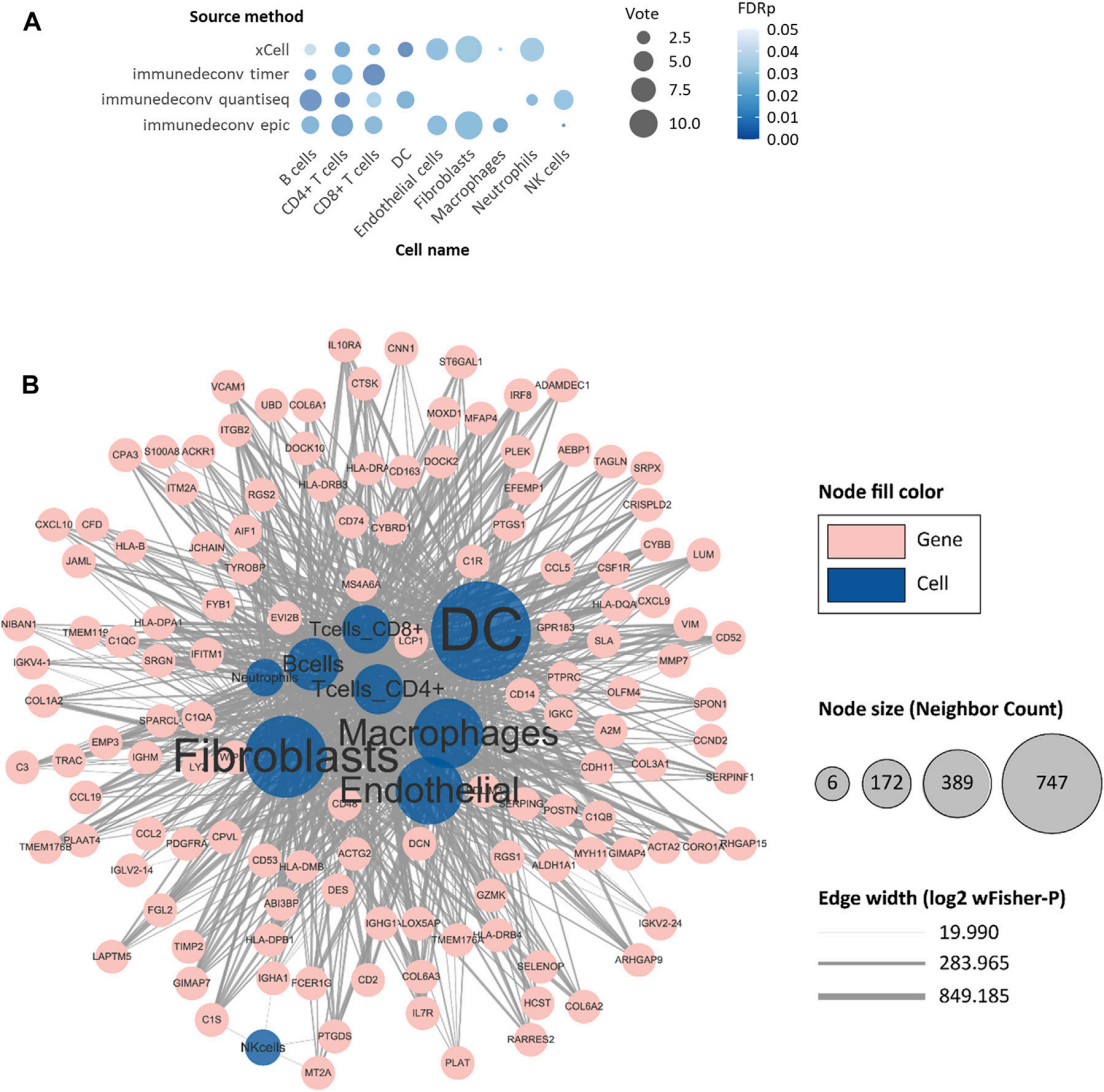
Identification of progression-risk-related tumor-infiltrating cells and differentially expressed genes representing them. **(A)** The non-weighted voting results of Student’s t-tests between tumor-infiltrating cells and well-established clinical progression risk factors. Tumor-infiltrating cell scores evaluated by six immune deconvolution methods were used. Only significant results were counted as valid votes shown in the figure. **(B)** The network of differentially expressed genes (DEGs) with their representing tumor-infiltrating cells. The blue circles refer to cell types. The pink circles refer to selected DEGs. The size of blue circles indicates the number of DEGs. The thickness of lines indicates the negative log2 of wFisher combined *p*-value of differential expression testing. Only nodes with more than six adjacent neighbors are shown.

Several risk factors have been proven to be significantly related to the prognosis of NMIBC patients (Liu et al., 2015; Douglas et al., 2021). Tumor size greater than 3 cm, multifocal lesions, concurrent CIS, more advanced cancer stage, higher histological grade, higher EORTC risk score, and higher frequency of prior recurrences were known risks implying higher rates of recurrence or progression. We first conducted a comparative analysis between these risk factors and 64 deconvoluted tumor-infiltrating cell types in each dataset, then employed an unweighted voting schema to identify top cell types that might contribute to NMIBC prognosis. As shown in Figure 2A, the top voted and most significant tumor-infiltrating cells included cancer-associated fibroblasts (CAFs), B cells, CD4^+^ T cells, CD8^+^ T cells, natural killer (NK) cells, dendritic cells (DCs), macrophages, neutrophils, and endothelial cells. Since xCell is typically used to determine the presence or absence of a specific cell type, rather than to calculate the cell proportion, we only used the sum of votes from the other five methods to filter the most relevant cell types (Supplementary Table S4). CD4^+^ T cells ranked first, being voted in five, three, and six of nine eligible datasets by TIMER (Li et al., 2016), quanTIseq (Finotello et al., 2019), and EPIC (Racle et al., 2017), respectively. Followed by CD4^+^ T cells, B cells, and CAFs.

### Biomarker Genes Representing the Candidate Tumor-Infiltrating Immune Cells

After targeting candidate tumor-infiltrating cells, we wished to ascertain a set of biomarker genes that were representative of the cells and that were also strongly associated with the survival of NMIBC patients. In identifying differentially expressed genes (DEGs) of the nine candidate immune cells, a total of 2757 “cell-DEG” pairs were recognized as repetitive patterns and included in the following analysis (Supplementary Table S6). We then analyzed all 972 nonredundant genes in the 2757 “cell-DEG” pairs with forest plot and Kaplan-Meier (KM) curve survival analyses against PFS in E-MTAB-4321, DFS in GSE32894, and OS in GSE13507 (Figure 2B). After this, we narrowed the list to 149 unique genes as protective or risk factors of PFS or OS in NMIBC patients. These genes with the cells they represented comprised 368 unique “cell-DEG” pairs (Table 2), of which 254 pairs were associated with PFS and 114 pairs with OS (Supplementary Table S7). DCs and CAFs were the top two cell types, with more than sixty percent (92/149, 91/149) of the biomarker genes associated with them (Table 2).

**TABLE 2:** List of biomarker genes representing the nine tumor-infiltrating candidate immune cells.

Bcells	DC	Endothelial	Fibroblasts	Macrophages	Neutrophils	NK cells	T cells_CD4+	T cells_CD8+
CD74	ADTRP	ADCY4	AKAP12	AP1S2	CD74	ANXA10	CASP1	CASP1
COL3A1	AP1S2	AP1S2	ANXA10	BTBD16	IGKV1-17	BTBD16	CD74	CD74
CXCL13	APOL3	BGN	AP1S2	C12orf75	MMP7	CLCA4	CFH	CXCL13
DES	ATF3	CD74	BGN	CAT	RARRES1	CRTAC1	COL1A1	DES
GIMAP7	ATP8B4	CLEC14A	BMP5	CD74	S100A8	ENTPD3	COL3A1	ENPP2
HCLS1	BMP5	CLIC4	BTBD16	CFH		FABP4	GIMAP7	FCER1A
IGHV1-69	CASP1	CLIP3	CCL11	CLIC4		FGFR3	GMFG	GDF15
IGKV1-17	CCL18	COL18A1	CD74	CNN3		RAB4A	IGKV1-17	GIMAP7
MMP7	CCL8	COL18A1	CLIC4	COL1A1		TMPRSS4	MMP7	IGKV1-17
POSTN	CD3G	COL1A1	CLIP3	COL3A1		TP63	MXRA5	SELENOP
RAC2	CD4	COL3A1	COL18A1	COL5A2			POSTN	SPINK1
RARRES1	CD74	COL4A1	COL18A1	CPQ			RAC2	SYNM
S100A8	CFH	COL4A2	COL1A1	CTSE			S100A8	TCF21
SELENOP	CLIC4	COL5A2	COL3A1	DEGS1			TRIM22	TRIM22
SERPINE2	CLIP3	COL8A1	COL4A1	DES			VCAN	
TRIM22	COL1A1	CRTAC1	COL4A2	DKK3			XAF1	
	COL3A1	CYGB	COL5A2	DOCK11				
	COL5A2	DEGS1	COL8A1	DSE				
	COL8A1	DES	CRTAC1	ELOVL5				
	CSF2RB	DKK3	CTSE	ENPP2				
	CSRP1	EDNRA	CXCL13	FBLN1				
	CXCL11	ENPP2	CYGB	FCER1A				
	CXCL13	FBLN1	DEGS1	FERMT2				
	DEGS1	FBN1	DES	FILIP1L				
	DES	FERMT2	DKK3	FSTL1				
	DKK3	FILIP1L	DOCK11	GIMAP7				
	DOCK11	FN1	DSE	GLT8D2				
	DSE	FSTL1	EDNRA	HCLS1				
	EDNRA	GEM	EFHD1	LITAF				
	ENPP2	GIMAP7	FABP6	LRIG1				
	FBN1	GLT8D2	FAM174B	MMD				
	FCER1A	GUCY1A1	FAM3B	MMP7				
	FERMT2	HCLS1	FBLN1	MXRA5				
	FGD2	ITGA1	FBN1	NUPR1				
	FGR	LAMA4	FCER1A	PLSCR4				
	FILIP1L	LRRC32	FERMT2	PODN				
	FN1	MFNG	FILIP1L	POSTN				
	FPR1	NEURL1B	FN1	PRDX3				
	FSTL1	NID1	FSTL1	RARRES1				
	GEM	NID2	GEM	RGS5				
	GIMAP7	NREP	GIMAP7	RPL17				
	GLT8D2	OLFML1	GLT8D2	S1PR3				
	GMFG	OLFML2A	GPX8	SELENOP				
	GPX8	PCDH17	GUCY1A1	SERPINE2				
	GUCY1A1	PDGFRB	HCLS1	SGCE				
	HCLS1	PLAC9	HOXB6	SH3BGRL				
	HLA-DQB2	PODN	IGFBP6	SLC9A9				
	HLA-E	POSTN	ITGA1	STEAP1				
	IGFBP6	PRRX1	LAMA4	SULF1				
	IGHV1-69	RBPMS2	LRIG1	SYNM				
	IGKV1-17	RGS5	LRRC32	TCF21				
	INPP5D	S100A8	MMD	TM4SF1				
	LAMA4	S1PR3	MMP7	TM4SF1				
	LITAF	SELENOP	MRVI1	TMED7				
	LRIG1	SERPINE2	MXRA5	TMEM45A				
	LRRC32	SGCE	NEURL1B	TNC				
	MAF	SULF1	NID1	TRIM22				
	MFNG	SYNM	NID2	TSPAN7				
	MMD	TCF21	NREP	VCAN				
	MMP7	TM4SF1	NUPR1	WDR72				
	MXRA5	TM4SF1	OLFML1					
	NEK6	TNC	OLFML2A					
	NUPR1	TSPAN7	PDGFRB					
	NXN	VCAN	PLAC9					
	OLFML1		PLN					
	PDGFRB		PLSCR4					
	PLSCR4		PODN					
	PLXDC2		POSTN					
	PODN		PRRX1					
	POSTN		RAC2					
	PRRX1		RBPMS2					
	RAC2		RGS5					
	S100A8		S100A8					
	SELENOP		S1PR3					
	SERPINA3		SELENOP					
	SERPINB9		SERPINA3					
	SERPINE2		SERPINE2					
	SGCE		SGCE					
	SP110		SMTN					
	SULF1		SULF1					
	SYNM		SYNM					
	TCF21		TCF21					
	TM4SF1		TEAD2					
	TM4SF1		TM4SF1					
	TMEM45A		TM4SF1					
	TNC		TMEM45A					
	TRIM22		TNC					
	TSPAN7		TPST1					
	VCAN		TSPAN7					
	XAF1		VCAN					
	ZFP36		VSIG2					
	ZG16B							

**FIGURE 3:**
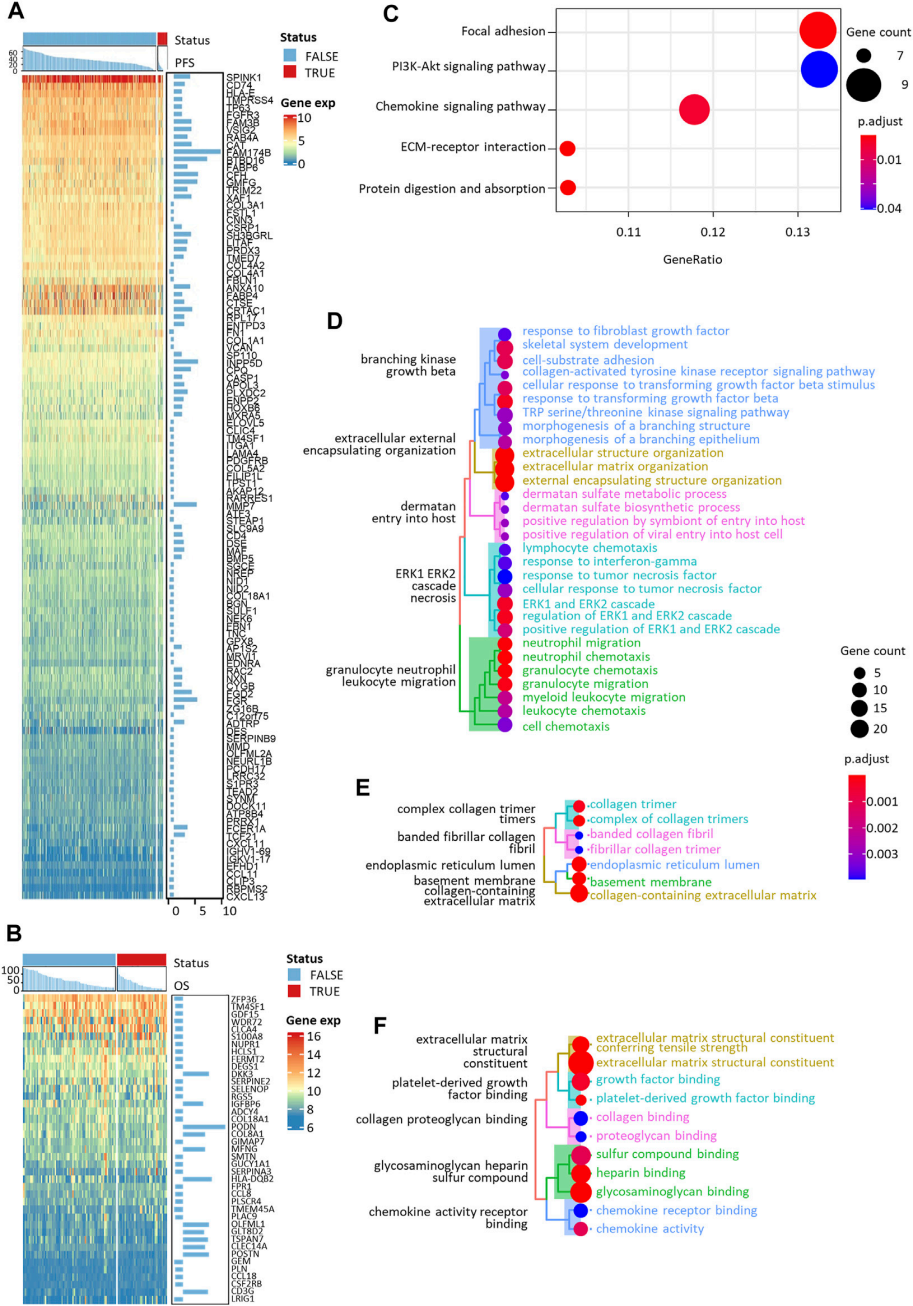
Expression heatmaps and functional enrichment analyses of PFS- and OS-related immune cell-specific DEGs. Expression heatmaps of **(A)** PFS-related and **(B)** OS-related DEGs. KEGG **(C)**, GO-biological process **(D)**, GO-cellular component **(E)**, and GO-molecular function **(F)** enrichment of all the selected DEGs.

The expression of 110 PFS-related and 41 OS-related biomarker DEGs was visualized in Figures 3A,B. All 99 biomarker DEGs of nine candidate tumor-infiltrating immune cells were subjected to KEGG pathway, GO-biological process (BP), GO-cellular component (CC), and GO-molecular function (MF) terms enrichment analyses (Figures 3C–F). As expected, we found strong evidence pointing to the crosstalk between tumor cells and adjacent normal epithelium, represented by focal adhesion and extracellular matrix (ECM)-receptor interaction. Aberration of these pathways would directly affect the steadiness of tumor cells and thereby cause progression. We also found enriched cell proliferation signals like protein digestion and absorption and the PI3K-Akt signaling pathway. They acted either as energy suppliers or as signal transduction factors to trigger or facilitate the cascade of invasive tumor progression. The chemokine signaling pathway, on the other hand, would help to recruit leukocytes to the site of the inflammation area.

### Enrichment of Tumor-Infiltrating Immune Cell Scores

**FIGURE 4:**
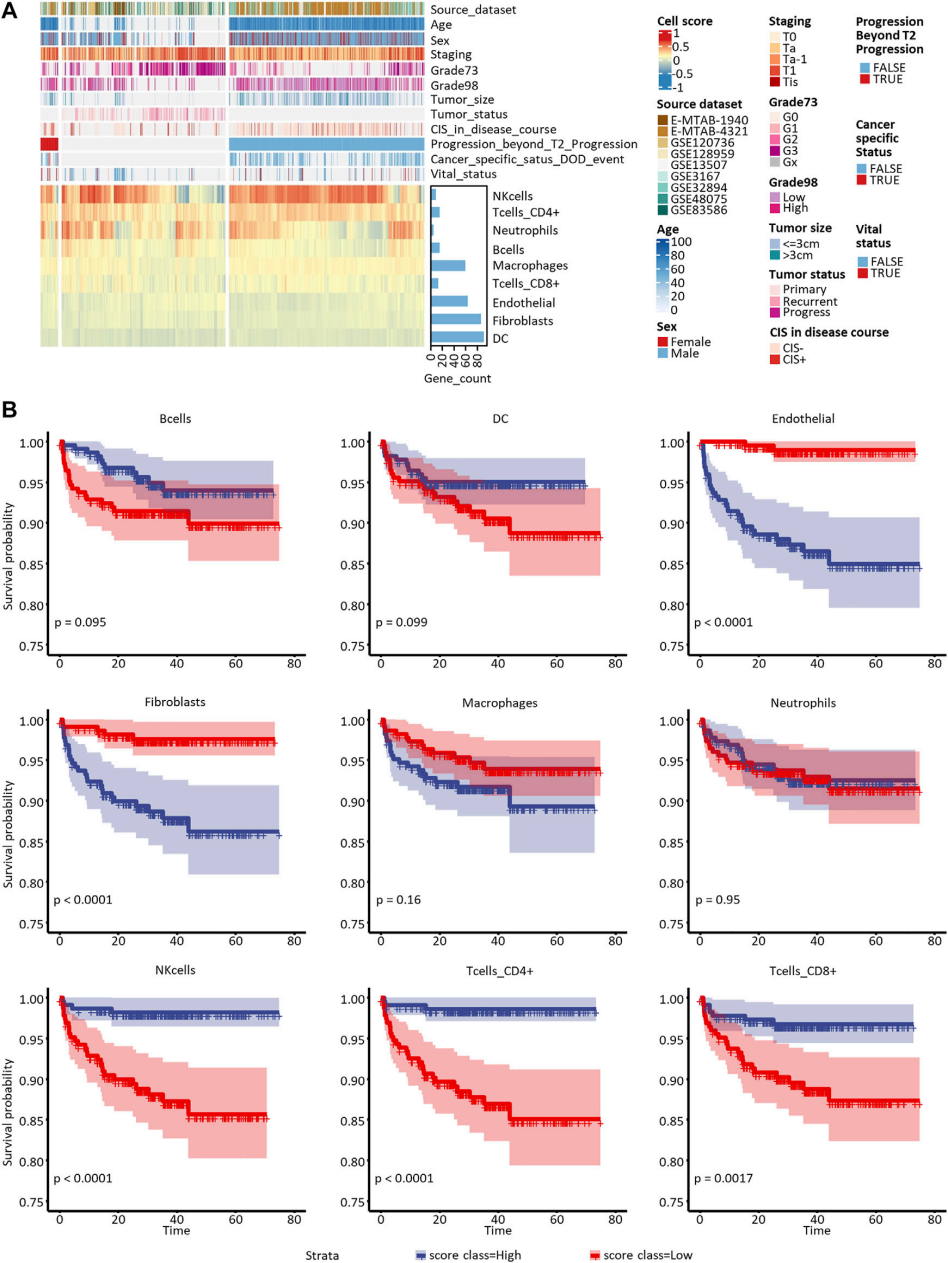
Proportion assessment and prognostic value of the nine candidate tumor-infiltrating cells. **(A)** Heatmap of candidate cells and clinical features of all the eligible 1,370 samples included in this study. Grade73 and Grade98 refer to the WHO 1973 and WHO 2004/2016 Classification Systems for Urothelial Carcinoma, respectively. **(B)** Kaplan-Meier curves of univariate Cox regression in low- versus high-infiltrated groups divided by the nine candidate immune or stromal cells.

Since the datasets included in our study differed in their transcriptome profiling technologies, we cautiously practiced the enrichment analyses with the logarithmic matrix of original expression data. 43,440 transcripts in 1,370 samples with and without log2-transformation were used to proceed with ssGSEA and z-score-based immune cell enrichment analyses. With the biomarker DEGs listed in Table 2 as priori-defined sets of immune cell-specific genes, we quantified the infiltration of all nine tumor-infiltrating immune cells in the tumor microenvironment. Enrichment of the cell scores by ssGSEA in all 1370 NMIBC transcriptomes is shown in Figure 4A.

To assess the nine immune cells’ ability to distinguish NMIBC patients with poor prognosis, we explored correlations between PFS, DFS, and OS with every tumor-infiltrating immune cell score calculated by ssGSEA and z-score methods. The survival analysis (Supplementary Table S8) showed that B cells, DCs, endothelial cells, CAFs, CD4^+^ T cells, and CD8^+^ T cells enriched by the ssGSEA method were significantly related to PFS in E-MTAB-4321 (Figure 4B). Macrophages and CD8^+^ T-cells enriched by the ssGSEA method were significantly related to OS in GSE13507 (Plots not shown). No cell types were significantly related to DFS in GSE32894.

### Robust Immuno-Prognostic Model

To achieve a robust prognostic model independent of the heterogeneous clinical information in eligible datasets, we used the score matrix of all nine candidate immune cells to build our model, although only some subsets of the cells were significantly related to PFS or OS. Since the primary goal of this study was to predict prognosis and risk of progression by key immune features, a total of 454 NMIBC samples from E-MTAB-4321 with assessable progression beyond T2 staging and PFS records were used. With the data, we repeatedly built training and test sets by randomly sampling 5000 times with a 1:1 ratio, fitted immune-prognostic models with the ridge regression, determined the optimal model with the minimum lambda, and evaluated the models with AUCs of ROC curves. Although immune cell enrichment score matrices calculated by both ssGSEA and z-score methods were used in building the immuno-prognostic model, only models built by ssGSEA matrices showed generally higher AUCs (data not shown). The formula of the final model was as follows:

Immuno-Prognostic score = - 0.4111588 + 2.5025813 * Bcells_score - 1.8274560 * DC_score + 6.7589250 * Endothelial_score + 2.6983895 * Fibroblasts_score - 0.1725197 * Macrophages_score + 1.0256969 * Neutrophils_score - 1.8221146 * NKcells_score - 6.0485265 * Tcells_CD4+_score—9.4937697 * Tcells_CD8+_score.

**FIGURE 5:**
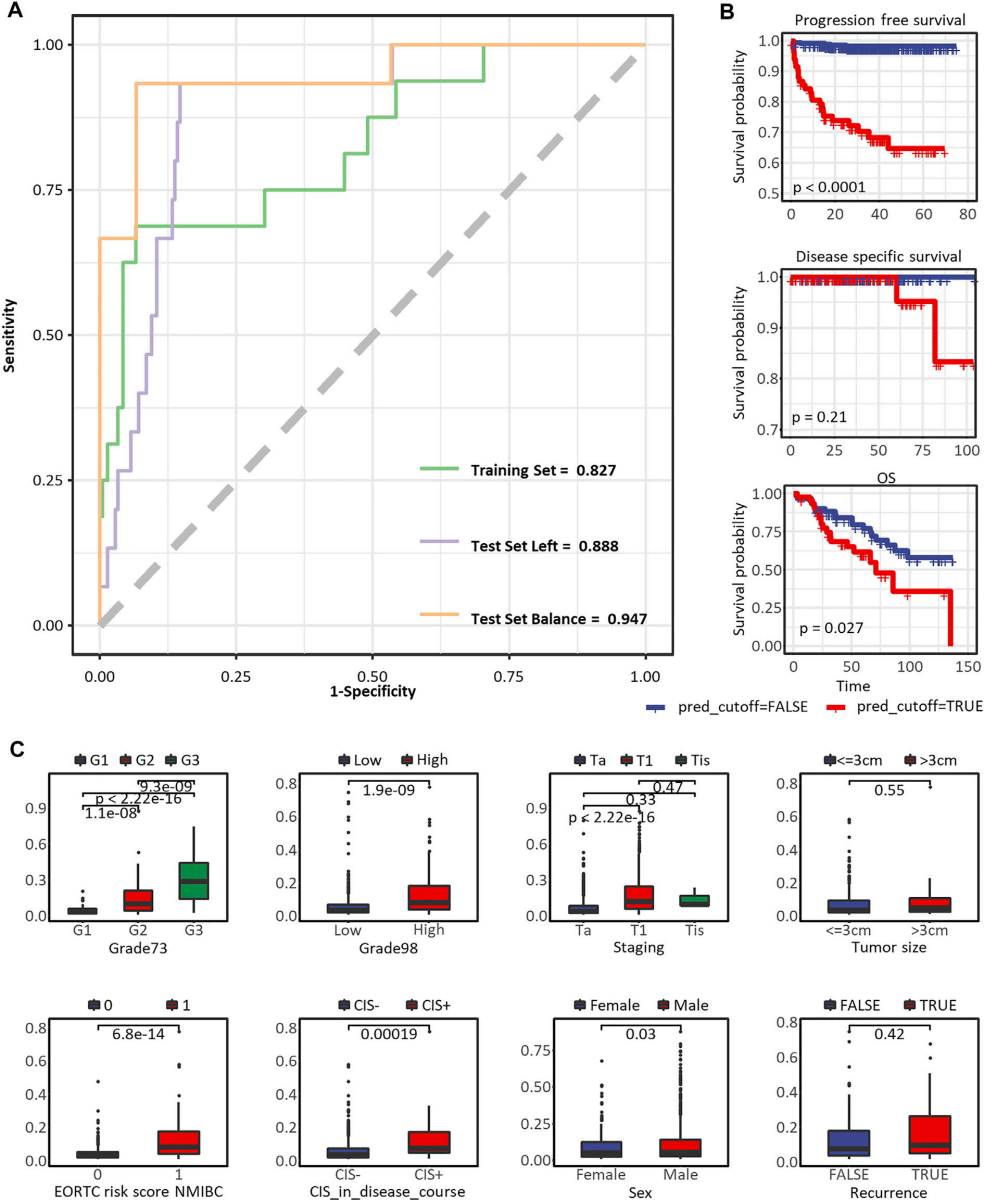
Predictive performance of the immuno-prognostic model. **(A)** The ROC curve to predict PFS in the training set, test set with all the other samples, and test set with balanced progressed and non-progressed samples. **(B)** The Kaplan-Meier curve to predict PFS, DFS, and OS*. **(C)** Box plots comparing risk scores assessed by the immuno-prognostic model in different groups of clinical prognostic risk factors. * In the nine eligible datasets, PFS status was assessed in E-MTAB-4321, GSE13507, and GSE32894, while only E-MTAB-4321 provided survival time. DFS status was assessed in GSE32894, GSE13507, and GSE48075, while only GSE32894 provided survival time. OS status was assessed in GSE13507 and E-MTAB-1940, while only GSE13507 provided survival time. As we plotted here, the survival analyses were only applicable to datasets E-MTAB-4321, GSE32894, and GSE13507.

We visualized the prediction effect of the optimal model in Figure 5A, the AUCs were 0.827, 0.888, and 0.947 in the training set (*n* = 228), test set with all the other samples (*n* = 226), and test set with balanced progression and non-progression patients (*n* = 30), respectively. The sampling groups of our optimal model are recorded in the last three columns in Supplementary Table S2. The optimal cutoff of the Immuno-Prognostic score dividing low-risk and high-risk patients was 0.109. In Figures 5A,B conspicuous differentiation of PFS (*p* < 0.0001, log-rank test) was observed in patients with different predicted outcomes. We also expanded our validation of the model in predicting other types of clinical outcomes. The same trend has been observed, but it showed less significance in predicting DFS (*p* = 0.21, log-rank test) and OS (*p* = 0.027, log-rank test). Furthermore, to test the correlation between our model and the well-established survival risk factors of NMIBC, we compared distributions of the predicted immuno-prognostic scores against different levels of CIS in the disease course, EORTC risk score, WHO 1973 or 2004/2016 grade, recurrence, sex, tumor stage, and tumor size. All comparisons showed higher immuno-prognostic scores in higher risk levels, but the trends were insignificant in recurrence status and tumor size. In summary, our model could predict the risk to the progression of NMIBC patients by evaluating the tumor-infiltrating microenvironment. The immuno-prognostic score well reflected the degree of progression risk.

## Discussion

With the assumption that cancer progression was associated with immune cell infiltrating, we performed an integrated analysis for developing a robust immuno-prognostic model to evaluate progression risk in NMIBC patients. We identified nine critical tumor-infiltrating cell types: innate immune cells including macrophages, neutrophils, DCs, and NK cells; adaptive immune cells including B cells, CD4^+^ T cells, and CD8^+^ T cells; and sentinel cells including CAFs and endothelial cells. The quantification of these immune cells was conducted by ssGSEA using the DEGs recognized from all eligible datasets. Univariate Cox regression supported that some cells could independently distinguish patients with high progression risk. Based on this, we achieved a more robust model using the enrichment matrix of all the nine tumor-infiltrating immune cells and then validated its performance in predicting different types of survival. The predicted risk scores and survival status showed a high correlation with the actual clinical outcomes; however, considering the precision and significance, we suggested using our model in predicting the PFS of NMIBC patients instead of DFS or OS.

We included nine immune cells in our model, even though some showed no independent prognostic value, since we thought their combination would better reflect the coordinated interaction between innate and adaptive immune systems in preventing the normal tissue from aggressive progression. For one thing, many genes were identified as the DEGs for more than one type of immune cells (Figure 2; Table 2); for another, the functional enrichment analysis of the full set of signature DEGs showed strong evidence of underlying drivers of tumor progression. The collagen family genes, for instance, were independently related to the survival of NMIBC and were simultaneously recognized as the DEG of tumor-infiltrating B cells, CD4^+^ T cells, CD8^+^ T cells, DCs, CAFs, macrophages, and endothelial cells. Xu and colleagues reviewed the mechanisms underlying this result (Xu et al., 2019). The complex reticular structure composed of collagen-rich extracellular matrices (ECM) and multiple stromal cells formed dense stromal fibrosis and thereby induced focal hypoxia, leading to increased tumor proliferation and compromised immunotherapy effectiveness (Daniel et al., 2019). The enriched KEGG pathways, including focal adhesion and ECM-receptor interaction (Figure 3C), were consistent with the previous description. The extensive interaction between stromal/immune cells and cancer cancers depicted the complexity of the tumor microenvironment, which was why we used cells instead of genes to build our model.

Another detail of our study was that we emphasized the selection of appropriate meta-analysis methods in the feature selection step and the careful use of renormalization methods. Toro-Domínguez and colleagues reviewed the three main types of meta-analysis strategies based on effect sizes, *p*-values combination, and rank combination (Toro-Domínguez et al., 2021). We chose wFisher (Yoon et al., 2021), a modified *p*-value combination method, to filter the DEGs representing candidate immune cells. The wFisher method was suitable for studies from different platforms or conditions. In our case, combining the analysis of nine transcriptomic datasets sequenced by both RNA-Seq and microarray platforms fit the method’s usage characteristics. The method also allowed combining results from heterogeneous analyses without rigorous renormalization. This feature elicited the second focus of our discussion: the renormalization of integrated transcriptomic data. Normalization of bulk RNA data included quantifying transcripts and standardizing data from different sources. The former was thoroughly discussed in the review of RNA sequencing technology (Stark et al., 2019). Here we mainly discussed the latter scenario, as the complexity of cancer biology required integrative studies with combined data from different researches. Shen and Wulff published their evaluations of various normalization methods for integrating large-scale metabolomics data, yielding the same conclusion that choosing the proper normalization method according to the data scale and downstream analysis would vastly improve the confidence of research results (Shen et al., 2016; Wulff and Mitchell, 2018). For transcriptome data, most studies still focused on the transcripts quantification question in the single-source dataset (Dillies et al., 2013; Li et al., 2015), while some of them also evaluated sophisticated frameworks and proposed a protocol to deal with raw RNA-Sequencing (RNA-Seq) data (Sahraeian et al., 2017). We found that few discussion has been made on the systematic renormalization of transcript data from multiple sources by multiple sequencing technologies, but some attempts were separately made and recommended in previous studies (Mooney et al., 2013; Risso et al., 2014; Ayers et al., 2017; Danaher, 2018; Liu et al., 2019). After modeling with both renormalized and non-normalized data (results shown in our Github or Gitee repositories listed in the Data Availability Statement section), we believed the renormalization method combining RNA-Seq and microarray data was still not well-established. We built our model for predicting PFS in NMIBC patients based on RNA-Seq data alone. We suggested that any further applications of our model should consider using RNA-Seq data rather than microarrays.

In conclusion, we identified nine critical tumor-infiltrating immune cells, quantified the cells’ proportion in the tumor immune microenvironment with self-defined signature genes, and established a robust immune-prognostic model for predicting the progression of NMIBC patients. Our study showed system-wide coordination of the immune and stromal cells in defending aberrant cell proliferation and aggressive tumor growth and invasion. Thus, modeling strategies regarding the tumor microenvironment as a whole system may be optimal in clinical decision support applications, which we believe is why multi-omics and integrative studies were replacing single biomarker and single dimension studies. In previous studies, single dimension data, such as the density of stromal TILs evaluated by H&E-stained slides, failed to predict survival outcomes independently (Rouanne et al., 2019; Ottley et al., 2020). Rouanne and colleagues only proved that the stromal TILs were associated with the tumor invasion depth in pT1 NMIBCs. Ottley and colleagues combined the sTILs levels with IHC and ISH biomarkers to improve the prognostic potential. In this shift to complex modeling with multiple dimension data, we raised the importance of appropriate data preprocessing procedures, including but not limited to the selection of appropriate meta-analysis methods. Moreover, some limitations of our research had to be mentioned here. With the inspiration from the UROMOL2021 study (Lindskrog et al., 2021), we initiated our investigation with the hypothesis that dynamic interactions in tumor immune microenvironment would reflect not only the progression risk but also the response to local treatment like intravesical instillation of chemotherapeutic or immunotherapeutic agents. Several efficient predictive biomarkers have been developed and widely evaluated in pan-cancer scenarios, such as the 18-gene gene expression profile (GEP) score (Ayers et al., 2017) has a high discriminatory value in predicting the response to pembrolizumab in Keynote-001, Keynote-012, and Keynote-028. Unfortunately, we did our research and failed to get enough high-quality response data to therapies in NMIBC patients. In the current study, we validated only the prognostic value of our model. Nevertheless, we wish to expand its usage in prognostic and predictive conditions in the future.

## Data Availability

Transcriptomic data of all datasets used in this study were available in public databases at the following URLs: E-MTAB-1940, microarray, ArrayExpress: https://www.ebi.ac.uk/arrayexpress/experiments/E-MTAB-1940/; E-MTAB-4321, RNA-Seq, ArrayExpress: https://www.ebi.ac.uk/arrayexpress/experiments/E-MTAB-4321/; GSE12073, microarray, GEO, https://www.ncbi.nlm.nih.gov/geo/query/acc.cgi?acc=GSE12073; GSE128959, microarray, GEO, https://www.ncbi.nlm.nih.gov/geo/query/acc.cgi?acc=GSE128959; GSE13507, microarray, GEO, https://www.ncbi.nlm.nih.gov/geo/query/acc.cgi?acc=GSE13507; GSE3167, microarray, GEO, https://www.ncbi.nlm.nih.gov/geo/query/acc.cgi?acc=GSE3167; GSE32894, microarray, GEO, https://www.ncbi.nlm.nih.gov/geo/query/acc.cgi?acc=GSE32894; GSE48075, microarray, GEO, https://www.ncbi.nlm.nih.gov/geo/query/acc.cgi?acc=GSE48075; GSE83586, microarray, GEO, https://www.ncbi.nlm.nih.gov/geo/query/acc.cgi?acc=GSE83586. All codes to reproduce the results and figures in this article and point-by-point responses were published on GitHub (https://github.com/XiaomengSun315/NMIBC_immuno-prognostic) repository.

## Abbreviations

AUA, American Urological Association; AUCs, areas under curves; BCG, Bacillus Calmette-Guerin; CAFs, cancer-associated fibroblasts; CIS or Tis, carcinoma *in situ;* DCs, dendritic cells; DEGs, differentially expressed genes; DFS, disease-free survival; EAU, European Association of Urology; ECM, extracellular matrix; EMT, epithelial-to-mesenchymal transition; EORTC, European Organisation for Research and Treatment of Cancer; FDR, false discovery rate; GEP, gene expression profile; GO, gene ontology; HRs, hazard ratios; IPS, immune prognostic signature; KEGG, Kyoto Encyclopedia of Genes and Genomes; MIBC, muscle-invasive bladder cancer; NK cells, natural killer; NMIBC, non-muscle-invasive bladder cancer; OS, overall survival; PFS, progression-free survival; ROCs, receiver operating characteristic curves; ssGSEA, single-sample gene set enrichment analysis; sTIL, stromal tumor-infiltrating lymphocyte; SUO, Society of Urologic Oncology; TIME, tumor immune microenvironment.
